# Reconsidering the Role of Cyclooxygenase Inhibition in the Chemotherapeutic Value of NO-Releasing Aspirins for Lung Cancer

**DOI:** 10.3390/molecules24101924

**Published:** 2019-05-18

**Authors:** Antonia Martin-Martin, Andrés Rivera-Dictter, Matías Muñoz-Uribe, Freddy López-Contreras, Jorge Pérez-Laines, Alfredo Molina-Berríos, Rodrigo López-Muñoz

**Affiliations:** 1 Instituto de Farmacología y Morfofisiología, Facultad de Ciencias Veterinarias, Universidad Austral de Chile, Valdivia 5090000, Chile; antoniamartinm94@gmail.com (A.M.-M.); ariveradictter@gmail.com (A.R.-D.); matias.munoz@uach.cl (M.M.-U.); freddy.lopez@postgrado.uach.cl (F.L.-C.); jorgeperezlaines@gmail.com (J.P.-L.); 2 Escuela de Graduados, Facultad de Ciencias Veterinarias, Universidad Austral de Chile, Valdivia 5090000, Chile; 3 Instituto de Investigación en Ciencias Odontológicas, Facultad de Odontología, Universidad de Chile, Santiago 8380492, Chile; aemolina@u.uchile.cl

**Keywords:** non-small-cell lung cancer, NCX4040, NCX4016, nitric oxide, erlotinib, prostaglandin, cyclooxygenase

## Abstract

Nitric oxide-releasing aspirins (NO-aspirins) are aspirin derivatives that are safer than the parent drug in the gastrointestinal context and have shown superior cytotoxic effects in several cancer models. Despite the rationale for their design, the influence of nitric oxide (NO**^•^**) on the effects of NO-aspirins has been queried. Moreover, different isomers exhibit varying antitumor activity, apparently related to their ability to release NO**^•^**. Here, we investigated the effects and mode of action of NO-aspirins in non-small-cell lung cancer (NSCLC) cells, comparing two isomers, NCX4016 and NCX4040 (*-meta* and *-para* isomers, respectively). NCX4040 was more potent in decreasing NSCLC cell viability and migration and exhibited significant synergistic effects in combination with erlotinib (an epidermal growth factor receptor inhibitor) in erlotinib-resistant cells. We also studied the relationship among the effects of NO-aspirins, NO**^•^** release, and PGE_2_ levels. NCX4040 released more NO**^•^** and significantly decreased PGE_2_ synthesis relative to NCX4016; however, NO**^•^** scavenger treatment reversed the antiproliferative effects of NCX4016, but not those of NCX4040. By contrast, misoprostol (a PGE_2_ receptor agonist) significantly reversed the antiproliferative effect of NCX4040, but not those of NCX4016. Furthermore, misoprostol reversed the antimigratory effects of NCX4040. Overall, these results indicate that PGE_2_ inhibition is important in the mode of action of NO-aspirins.

## 1. Introduction

Lung cancer is a global threat and had the highest estimated incidence and mortality rate of all cancers in 2018 [1]. As most newly diagnosed patients with non-small-cell lung cancer (NSCLC) are at an advanced stage of the disease, the goals of treatment are to improve survival and reduce disease-related events. Current chemotherapy for this malignancy involves the use of cytotoxic drugs, alone or in combination, including platinum derivatives (cisplatin or carboplatin) [2] and antimetabolites (i.e., pemetrexed) [3]. For patients with a specific mutation in the epidermal growth factor receptor (EGFR), the use of tyrosine kinase inhibitors such as erlotinib is recommended [4]. However, all the drugs used are associated with a variety of adverse effects, and no chemotherapy strategy is proven to increase survival over 10 months for patients with advanced stage disease [5].

Research aimed at identifying safer cancer treatments has suggested that non-steroidal anti-inflammatory drugs (NSAIDs), such as acetylsalicylic acid (aspirin), sulindac, and indomethacin have chemopreventive and antitumor effects against several types of malignancy, including colorectal, ovarian, breast, and lung cancer [6,7,8,9]; however, continuous NSAID use is strongly associated with gastric ulcers (induced by non-selective NSAIDs) or cardiovascular effects (caused by cyclooxygenase-2 selective inhibitors) [10,11]. Given these adverse effects, novel derivatives, such as nitric oxide-releasing NSAIDs (NO-NSAIDs), have been developed by adding a nitro group, able to release a nitric oxide (NO**^•^**) molecule, to widely used NSAIDs, including aspirin, flurbiprofen, or diclofenac. These NO-NSAIDs have been proven to be safer at the gastrointestinal level, while maintaining their anti-inflammatory proprieties [12,13]. Moreover, the NO**^•^** released by NO-NSAIDs acts as a cardiovascular modulator, improving the cardiovascular benefits of NSAIDs, including aspirin [14].

In addition to their gastric safety and cardiovascular effects, NO-NSAIDs may also have roles in cancer treatment or chemoprevention. In this context, NO-NSAIDs derived from aspirin, sulindac, and ibuprofen showed superior antiproliferative effects compared with their parental NSAIDs in lung, pancreas, colon, prostate, and tongue tumor cells lines [15]. Furthermore, NO-sulindac has shown chemopreventive effects in an animal model of ultraviolet-B-induced skin cancer [16] and antiproliferative effects on several bladder carcinoma cell lines [17]. In addition, NO-indomethacin significantly reduced the incidence and invasiveness of tumors in a drug-induced colon cancer model [18], and NO-ibuprofen induced apoptotic cell death via caspase activation in prostate cancer cells [19].

**Figure 1:**
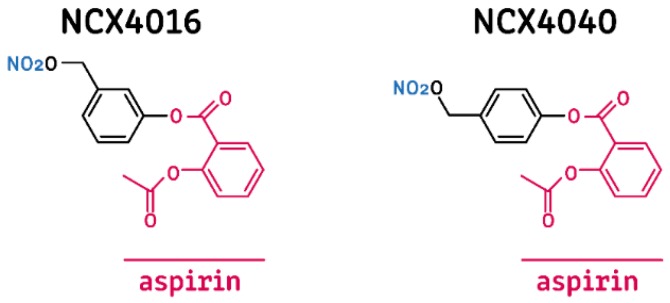
Chemical structure of the NO-aspirins used in this study. The structure of aspirin is highlighted in red, and the nitro (nitric oxide-releasing) group is shown in blue. The linker group is shown in black.

To date, the most studied NO-NSAIDs are those derived from aspirin (NO-aspirins), namely NCX4060, NCX4040, and NCX4016, which possess an –ONO_2_ substituent covalently linked through a benzene mediator in the *-ortho*, *-meta*, or *-para* position (relative to the carboxylic group of aspirin), respectively (Figure 1) [20]. NCX4016 is reported to be effective in both in vivo and in vitro models of leukemia, ovarian cancer, and colon cancer [18,21,22,23,24], while NCX4040 has been evaluated in colon and pancreatic cancer models [25,26,27,28]. NCX4060 is the least studied of this group of compounds and has shown antiproliferative effects against prostate cancer and non-small-cell lung cancer (NSCLC) cells [19,29]. There is also evidence that NCX4060 can induce apoptosis in NSCLC by disrupting the EGFR-AKT pathway [29].

Despite the rationale behind the design of NO-aspirins, their modes of action remain incompletely elucidated. There is evidence that opposes the role of nitric oxide in the antitumoral activity of NO-aspirins, and points to the disruption of the “linker” moiety, yielding the formation of a quinone intermediate and release of salicylic acid [30,31].

Among the three known NO-aspirins, NCX4040 and NCX4016 are the best studied; however, neither their antiproliferative and antimigratory activities in NSCLC models nor their mode of action in this type of tumor cell have yet been addressed. Thus, the aims of this study were to evaluate the antiproliferative and antimigratory effects of NCX4040 and NCX4016 in NSCLC cells, investigate their interactions with the EGFR inhibitor, erlotinib, and explore the relationship among NO• release, prostaglandin E_2_ (PGE_2_) synthesis inhibition, and the effects of these NO-aspirins.

## 2. Results

### 2.1. NO-Aspirins Reduce NSCLC Cell Viability and Migration with Different Potencies

We produced concentration–response curves using MTT reduction and BrdU uptake assays as indirect measures of NSCLC cell viability and proliferation, respectively. The results are shown in Figure S1 and summarized in Table 1. In both assays, NO-aspirins had more potent effects than aspirin, the parental drug. The EC_50_ values for both NCX4016 and NCX4040 were significantly lower than that of aspirin (approximately 10- and 100-fold, respectively).

**Figure 2:**
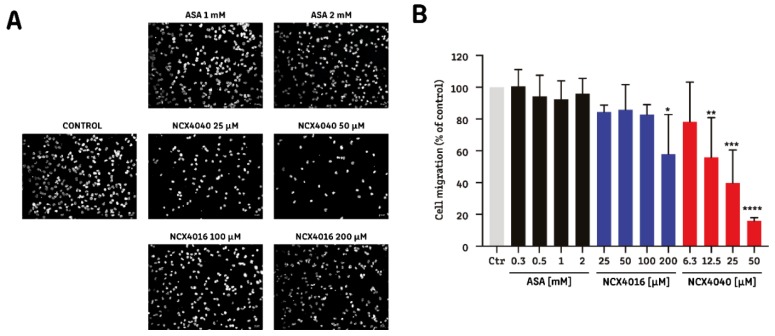
Effect of NO-aspirins on non-small-cell lung cancer cell migration. Assays were conducted by evaluating the migration of H1299 cells through an 8 μM-pore cell culture insert. Cells were stained with DAPI after 6 h of migration. (**A**) Representative images of migration of H1299 cells exposed to aspirin (ASA; 1 and 2 mM), NCX4016 (100 and 200 μM), and NCX4040 (25 and 50 μM). (**B**) Quantitation of migration of H1299 cells exposed to aspirin (ASA; from 0.3 to 2 mM), NCX4016 (from 25 to 200 μM), and NCX4040 (from 6.3 to 50 μM). The bar graph summarizes the results from four independent experiments. * *p* < 0.05; ** *p* < 0.01; *** *p* < 0.001, and **** *p* < 0.0001, compared with the control group, calculated by one-way ANOVA and Dunnett’s post-test.

In addition, we measured the migration ability of H1299 cells after exposure to the drugs for 6 h. As shown in Figure 2, aspirin did not reduce cell migration at the assayed concentrations; however, NCX4016 at the highest concentration (200 µM) and NCX4040 at concentrations ≥ 12.5 µM, significantly reduced cell migration compared with untreated controls.

### 2.2. NCX4040 and Erlotinib Have Synergistic Effects on NSCLC Cell Lines

Erlotinib is a tyrosine kinase inhibitor used as a targeted therapy against EGFR-mutated NSCLC. To study the effects of combinations of NO-aspirins and erlotinib, we used six concentrations of each drug, and every possible combination of them, in a chess-matrix scheme and analyzed the resulting data using the Loewe additivity model, with the free software COMBENEFIT [32]. Erlotinib is effective regardless of the presence or absence of the EGFR mutation; however, it is more potent in cells carrying the mutation. Thus, we performed a comparison of the drugs’ effects over a panel of three NSCLC cell lines. We used the H1299 and A549 cell lines, which are wild-type for the EFG receptor, and H1975 cells, which carry the T790M and L858R mutations in the EGF receptor [33]. We compared EC_50_ values of the studied drugs in the three cell lines. The EC_50_ value for erlotinib in H1975 cells was 4.77 µM, compared with 8.22 µM in A549 cells. By contrast, A549 cells were significantly more resistant to treatment with NCX4040 than H1975 cells, with EC_50_ values of 23.8 and 7.0 µM, respectively (Table 2 and Figure S2). When the combination analysis was performed (Table 3 and Figure S3), NCX4040 showed the greatest synergistic effect when combined with erlotinib in two of the three NSCLC cell lines assayed, with a synergy score of 20 ± 3 for A549 cells and 13 ± 3 for H1299 cells. It is important to note that greater synergistic effects were observed when NCX4040 and erlotinib were used at higher concentrations (25 µM of NCX4040 and 50 µM of erlotinib, Figure S3). Interestingly, a synergistic effect was observed in the cell types that were more resistant to the effects of erlotinib (A549 and H1299). No synergistic effects of aspirin were detected in any cell type, even at extremely high concentrations (e.g., 5 mM).

### 2.3. The Effect of NCX4016, but Not That of NCX4040, Is Related to Its Ability to Release NO**^•^**

**Figure 3:**
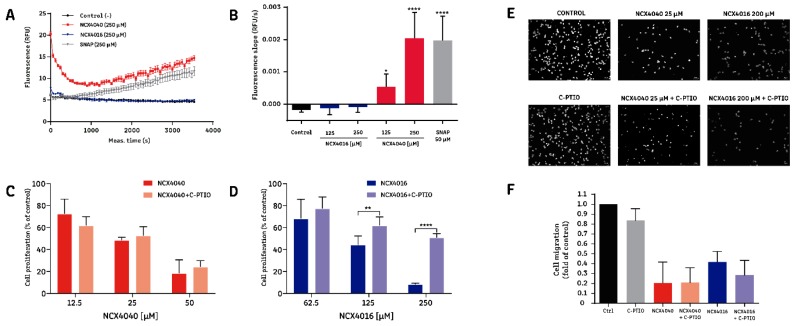
The relationship between nitric oxide (NO**^•^**) release by NO-aspirins and their effects on non-small-cell lung cancer (NSCLC) cells. Intracellular NO**^•^** release by NO-aspirins was measured using a DAF-2 fluorescent probe. H1299 cells were pre-loaded with DAF-2 prior to incubation in culture plates. After 24 h, cells were exposed to the different drugs. Cell viability was measured by MTT reduction. Cell migration was evaluated using an 8 µM-pore cell culture insert and fluorescence microscopy. (**A**) Representative graph showing the relative fluorescence induced by NO**^•^** release from NO-aspirins. (**B**) Slope of fluorescence over time, induced by NO**^•^** release from NO-aspirins. SNAP was used as a positive control for NO**^•^** release. * *p* < 0.05 and **** *p* < 0.00001, compared with the negative control, calculated by one-way ANOVA and Dunnett’s post-test. (**C**) Cell proliferation after exposure of H1299 cells to NCX4040 and NCX4040 + C-PTIO for 96 h. (**D**) Cell proliferation after exposure of H1299 cells to NCX4016 and NCX4016 + C-PTIO for 96 h. ** *p* < 0.01 and **** *p* < 0.00001, between the indicated groups, calculated by one-way ANOVA and Tukey post-test. (**E**) Representative images of H1299 cell migration following exposure to NO-aspirins alone or in the presence of C-PTIO. (**F**) Quantitation of migration of H1299 cells exposed to NO-aspirins alone and in the presence of C-PTIO. Bar graphs summarize the results of at least three independent experiments.

We evaluated NO**^•^** release in H1299 cells exposed to aspirin, NCX4016, and NCX4040. As shown in Figure 3, NCX4040 was the only molecule that released NO**^•^** in detectable amounts (Figure 3A,B). Furthermore, we explored whether the effects of NO-aspirins were associated with NO**^•^** release. To this end, we repeated the cell viability and migration experiments in the presence of the NO**^•^** scavenger, carboxy-PTIO (C-PTIO). C-PTIO (10 µM) reversed the effects of NCX4016 on cell viability, whereas the effects of NCX4040 were not influenced by C-PTIO (Figure 3C,D). By contrast, evaluation of cell migration (Figure 3E,F) demonstrated that NCX4016 (200 µM) and NCX4040 (25 µM) significantly reduced migration, and that this effect was not modified by C-PTIO. Together, these results suggest that the effects of NCX4016, but not those of NCX4040, are at least partially dependent on its ability to release NO**^•^**. Nevertheless, the effects of NO-aspirins on cell migration may be attributable to a different mechanism of action.

### 2.4. The Effect of NCX4040 is Related to Its Ability to Inhibit PGE_2_ Synthesis

**Figure 4:**
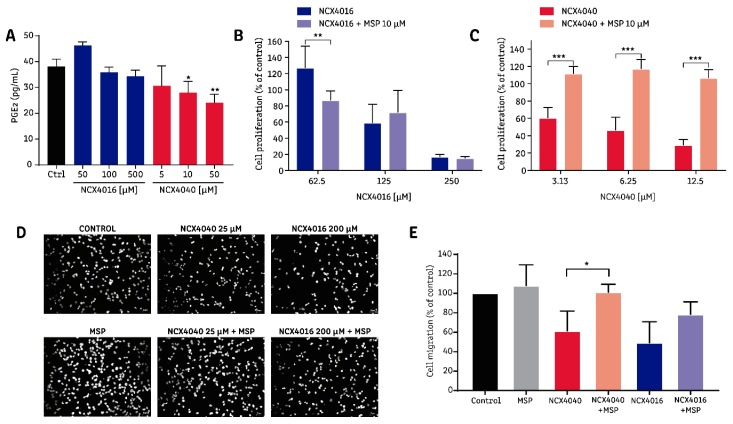
Relationship between the inhibition of prostaglandin E_2_ (PGE_2_) synthesis by NO-aspirins and their effects on non-small-cell lung cancer (NSCLC) cells. PGE_2_ levels were measured in H1299 cells exposed to NO-aspirins for 24 h. To evaluate cell viability, cells were exposed to NO-aspirins or NO-aspirins + misoprostol (MSP) for 96 h, then cell viability was measured by MTT reduction. Cell migration was evaluated after 6 h of exposure using an 8 μM-pore cell culture insert and fluorescence microscopy. (**A**) PGE_2_ levels in the supernatant of H1299 cells. * *p* < 0.05; ** *p* < 0.01, and *** *p* < 0.0001, compared with the control group, calculated by one-way ANOVA and Dunnett’s post-test. (**B**) Cell viability of H1299 exposed to NCX4016 or NCX4016 + MSP for 96 h. (**C**) Cell viability of H1299 cells exposed to NCX4040 or NCX4040 + MSP for 96 h. (**D**) Representative images of H1299 cell migration following exposure to NO-aspirins alone or in the presence of MSP. (**E**) Quantitation of the migration of H1299 cells exposed to NO-aspirins alone and in the presence of MSP. Bar graphs summarize the results of at least three independent experiments. For panels (B), (C), and (E), * *p* < 0.05; ** *p* < 0.01, and *** *p* < 0.0001, between the indicated groups, calculated by one-way ANOVA and Tukey post-test.

The rapid release of NO**^•^** by NCX4040 requires an initial deacetylation of NO-aspirin, generating salicylic acid, which can inhibit the COX enzyme [20]. A logP calculation, performed using the Chemicalize^®^ online tool (https://chemicalize.com), indicates that NO-aspirins are more liposoluble than aspirin. NO-aspirins have a logP value of 3.17, which does not change with pH condition (logD), as NO-aspirins do not contain any ionizable groups. By contrast, aspirin has a logP value of 1.27; however, at physiological pH (7.4) its coefficient of partition (logD) falls to −2.7, decreasing its lipid solubility. Thus, the higher lipid solubility of NCX4040, in addition to its rapid intracellular deacetylation, suggests that increased intracellular salicylic acid is likely to be available to inhibit COX when this drug is applied. Therefore, we evaluated PGE_2_ production in H1299 cells exposed to NO-aspirins and found that NCX4040 could significantly reduce PGE_2_ synthesis at 10 and 50 µM (Figure 4A). Misoprostol (10 µM), a prostaglandin receptor agonist, completely abolished the antiproliferative effect of NCX4040, but not that of NCX4016, suggesting that the inhibition of PGE_2_ synthesis by COX may have a pivotal role in the antiproliferative effect of NCX4040 (Figure 4B,C). When we analyzed the effect of misoprostol on the antimigratory proprieties of NO-aspirins, we found that misoprostol significantly reversed the antimigratory effect of NCX4040, and tended to reverse the effect of NCX4016, but without statistical significance (Figure 4D,E).

## 3. Discussion

A stronger antiproliferative effect of NCX4040 compared with aspirin has been previously reported in colon, prostate, tongue, pancreas, and lung cancer cells lines [15,34]. In lung cancer cells, NCX4040 is reported to have an EC_50_ value 50-fold lower than that of aspirin [15], consistent with our data, which indicates aspirin to NCX4040 EC_50_ ratios of 69 and 324.8, based on analyses of cell viability (determined by MTT reduction assay) and cell proliferation (demonstrated by BrdU uptake), respectively.

Comparisons between NO-aspirin isomers have also been performed in HT-29 colon cancer cells, in which NCX4040 and NCX4060 are approximately 100 and 200 times more potent, respectively, than NCX4016. This difference remained when NCX4016 and NCX4040 were assayed using an in vivo model of colon cancer [35]. Furthermore, NCX4040 has superior activity compared with NCX4016 in two ovarian cancer cell lines: A2780 and A2780-cDDP (cisplatin-resistant) [23]. Here, we report the first comparison of two NO-aspirins in NSCLC cells; our data agree with previous reports showing that NCX4040 is more potent than NCX4016 with regard to its effects on cell viability, proliferation, and migration (Table 1, Figure S1 and Figure 2). There have been no previous reports about the antimigratory proprieties of NO-aspirins. We found that NCX4040 also has superior antimigratory effects on NSCLC cells compared with NCX4016 and aspirin.

NCX4060, the *-ortho* isomer of NCX4040, was previously reported to increase the effects of the EGFR inhibitor, erlotinib [29]. In agreement with these data, our study demonstrates that the A549 and H1299 cell lines (wild-type for EGFR [36]) are more resistant to erlotinib and NCX4040 than the H1975 EGFR-mutant cell line (Table 2 and Figure S2). Notably, the previous study demonstrated only the combined effects of NCX4060 in erlotinib-sensitive cell lines. Here, we show that the synergism between NCX4040 and erlotinib is stronger in the resistant cell lines, A549 and H1299 (Table 3 and Figure S3), suggesting that this combination strategy could also be useful in the absence of a specific EGFR mutation.

Previous reports indicate that the differences in potency of NO-aspirins against colon cancer cells correlate with their varying ability to release NO**^•^** [20,35]. Our data show that NO**^•^** can be detected in the intracellular milieu when cells are exposed to NCX4040, but not NCX4016; however, when the NO**^•^** scavenger C-PTIO was added, only the effects of NCX4016 were significantly inhibited (but not abolished). These apparently contradictory results could be explained by the different intracellular metabolic processes undergone by these molecules. After a rapid enzymatic deacetylation and cleavage of the ester bond, NCX4040 releases a NO_3_^−^ group (that finally converts to NO**^•^**). By contrast, NCX4016 generates a stable (nitroxymethoxy) phenol, which slowly generates NO_3_^−^ and, consequently, NO**^•^** [20]. Thus, it is likely that the time during which NO**^•^** release was measured (1 h) was too short to detect NO**^•^** release by NCX4016; however, the reversion caused by C-PTIO (measured as cell viability after 96 h) indicates that NO**^•^** does have a role in mediating the effects of NCX4016. Moreover, the lack of C-PTIO-induced reversion of the effects of NCX4040 does not negate the potential role of NO**^•^**. Indeed, it is possible that C-PTIO cannot stoichiometrically react and neutralize the quantity of NO**^•^** released by the extremely fast and extensive process that occurs after NCX4040 exposure. Unfortunately, higher concentrations of C-PTIO cannot be used, as they influence cell viability. Also, it has been suggested that the effects of these NO-aspirins are attributable to the formation of a quinone methide (QM) metabolite, which is released during the same process that causes NO**^•^** release. The QM metabolite can react with cellular thiols, such as glutathione (GSH), and macromolecules, such as DNA [20,30,31,37].

The effect of NO-aspirins on PGE_2_ synthesis have been reported previously. In vitro, NCX4016 (300 µM) significantly reduced PGE_2_ production in human monocytes, and this effect was partially associated with NO**^•^** production, via guanylyl-cyclase (GC) activity [38]. Also, studies using isolated COX-1 enzyme demonstrated its direct irreversible inhibition by NCX4016, presumably via acetylation of the COX-1 enzyme [38]; however, direct inhibition by COX enzyme acetylation seems improbable in vivo, owing to the rapid metabolism of NXC4016 to salicylic acid, as evidenced in a study using rat liver microsomes [39] and in humans, where a treatment with NCX4016 led to the formation of salicylic acid as the main metabolite [40]. By contrast, NCX4040 also decreased PGE_2_ levels in whole human blood, but this effect was not reversed by a GC inhibitor, suggesting that a mechanism other than the activation of the NO**^•^**-GC axis must be involved [41]. The relationship between the antitumor effects of NCX4040 and COX inhibition has been studied in colon cancer cells, showing that colon cancer cell lines overexpressing the COX-2 isoform were more resistant to the cytotoxic effect of NCX4040; surprisingly, in those cells exhibiting resistance to the effects of NCX4040, there was an increase in PGE_2_ following exposure to NCX4040 [25,26]. Despite our data of lung cancer cells showing that NCX4040 significantly decreases PGE_2_ levels after 4 h of exposure, the EC_50_ values obtained in three different NSCLC lines (Figure 3, lower row) are consistent with previous data, as the two more resistant cell lines (A549 and H1299) expressed higher levels of COX-2 than the H1975 cell line [42]. Importantly, this is the first report showing that a prostaglandin receptor agonist (misoprostol) can abolish the antiproliferative effects and reverse the antimigratory effects of NCX4040.

Among the four prostaglandin receptor types, EP1, EP2, and EP4 have important roles in the survival and migration of several cancer types [43]. Misoprostol behaves like an agonist of the EP2, EP3, and EP4 receptors, suggesting that the decreased levels of PGE_2_ induced by NCX4040 could impair signaling via these two types of receptor. Accordingly, the EP2 and EP4 receptors have proven to be important for both the proliferation and migration of H1299 and A549 cells [42].

Overall, this report shows that the NO-aspirin isomers NCX4016 and NCX4040 have differential effects derived from their varying abilities to release NO**^•^** and decrease PGE_2_ levels. Regarding their antiproliferative effects, that of NCX4016 is related to NO**^•^** release, whereas that of NCX4040 is strongly associated with its ability to decrease PGE_2_ production; nevertheless, PGE_2_ inhibition seems to be important for the antimigratory effects of both drugs.

## 4. Materials and Methods

### 4.1. NSCLC Cell Culture

The human NSCLC cell lines H1299 (ATCC^®^ CRL-5803™), A549 (ATCC^®^ CCL-185™), and H1975 (ATCC^®^ CRL-5908™) were purchased from ATCC (Manassas, VA, USA). Cells were cultured in humidified air with 5% CO_2_, using RPMI 1640 (for H1299 and H1975 cells) or DMEM F-12K (for A549 cells) culture media, supplemented with 10% fetal bovine serum (FBS) and antibiotics (penicillin, 100 U/mL and streptomycin, 100 μg/mL). All cells were used for up to 20 passages.

### 4.2. Drugs and Experimental Design

The drugs used in this study were: aspirin (Sigma-Aldrich, St. Louis, MO, USA), NCX4040 (Tocris Bioscience, Bristol, UK), NCX4016 (USBiological, Salem, MA, USA), and erlotinib (Cayman Chemical, Ann Arbor, MI, USA). Alternatively, cells were preincubated with Carboxy-PTIO, potassium salt (Sigma-Aldrich, St. Louis, MO, USA) as an NO**^•^** scavenger. Misoprostol (Sigma-Aldrich, St. Louis, MO, USA) was used as a prostaglandin-receptor agonist. All drugs were dissolved in DMSO, with the exception of misoprostol, which was dissolved in ethanol. For all experiments, the maximal concentration of vehicle per well was 0.05%, which did not influence cell viability. For cell viability studies, cells were exposed for 96 h to seven concentrations of each drug, in two-fold dilution series from 5 mM, 250 µM, or 50 µM of aspirin, NCX4040, or NCX4016, respectively. For cell proliferation experiments, cells were exposed for 24 h to five concentrations of each drug, in two-fold dilution series from 5 mM, 500 µM, or 25 µM of aspirin, NCX4040, or NCX4016, respectively. For cell migration experiments, we assayed four concentrations of each drug in two-fold dilution series from 2 mM, 200 µM, and 50 µM of aspirin, NCX4016, and NCX4040, respectively. All these concentration ranges were consistent with those used in previous studies using the same drugs to treat in vitro models of cancer [31,34].

### 4.3. Evaluation of Cell Viability by MTT Reduction

NSCLC cells were seeded in 96-well plates at densities of 2000, 2500, or 5000 cells per well for the A549, H1299, and H1975 cell lines, respectively. Cells were allowed to attach the wells for 24 h before drugs were added. After drug exposure for 96 h, cells were incubated with MTT (Sigma-Aldrich, St. Louis, MO, USA) at 0.5 mg/mL for 4 h. The formazan crystals formed were solubilized with 100 µL of 10% sodium dodecyl sulfate in 0.01 M HCl by overnight incubation at 37 °C. Formazan production was measured at 579–690 nm in a Varioskan plate reader (Thermo Scientific, Waltham, MA, USA) [44].

### 4.4. Evaluation of Cell Proliferation by Uptake of 5-Bromo-2’-Deoxyuridine (BrdU)

BrdU uptake was detected using the BrdU Cell Proliferation Assay ELISA kit (Merck Millipore, Darmstadt, Germany), following the manufacturer’s instructions. Briefly, H1299 cells were seeded in 96-well plates at a density of 2500 cells per well and allowed to attach overnight. Then, cells were exposed to the different drugs and, after 24 h, 20 µL of 500× BrdU solution was added, with 200 µL of fixing solution added after a further 24 h, followed by incubation at 37 °C for 30 min. Plates were then washed and 100 µL of anti-BrdU primary antibody was added, followed by incubation for 1 h, washing, and addition of 100 µL secondary antibody and incubation for 30 min. Then, plates were washed, 100 µL of substrate solution was added and, after 30 min, 100 µL of stop solution was added. Plates were read at 450 nm on a Varioskan plate reader (Thermo Scientific, Waltham, MA, USA).

### 4.5. Measurement of Nitric Oxide (NO**^•^**) Release

H1299 cells were incubated in white 96-well plates, at a density of 10,000 cells per well, in RPMI media (without FBS) with 10 µM 4,5-diaminofluorescein diacetate (DAF-2DA) for 1 h at 37 °C. Cells were then centrifuged at 1000 *g* for 8 min, resuspended in complete medium, seeded in 96-well plates, and allowed to attach for 24 h. Cells were exposed to drugs under standard culture conditions and in darkness. Fluorescence was evaluated by 1 h kinetics, using excitation and emission wavelengths of 485 and 538 nm, respectively.

### 4.6. Prostaglandin E_2_ Measurement

Prostaglandin E_2_ (PGE_2_) production was evaluated using the Prostaglandin E_2_ ELISA kit—monoclonal (Cayman Chemical, Ann Arbor, MI, USA), following the manufacturer instructions. Briefly, cells were seeded in 24-well plates at a density of 200,000 cells per well and allowed to attach for 24 h. Subsequently, cells were exposed to the different drugs for 24 h. Then, supernatants were transferred to clear 1.5 mL tubes and centrifuged at 8000 *g* for 10 min at 4 °C. Aliquots (50 μL) of each sample were added to the IgG coated plate, and 50 μL of the PGE_2_ AChE added to each well, with the exception of the total activity and blank wells. Then, 50 μL prostaglandin E_2_ monoclonal antibody was added to each well, except for the total activity, blank, and non-specific binding wells. Subsequently, the plate was incubated for 18 h at 4 °C. The plate was then washed five times and Ellman’s reagent (200 μL/well) added, followed by incubation of the plate in the dark for 1 h at room temperature. Finally, the color developed was measured in a Varioskan plate reader (Thermo Scientific, Waltham, MA, USA) at 410 nm.

### 4.7. Cell Migration

Cell migration was assessed using an 8 µm-pore cell culture insert (Corning Life Sciences, NY, USA). H1299 cells were seeded in the upper part of the insert, in RPMI medium without serum. In the lower part of the insert, 300 µL of complete media was used as a chemoattractant. After 6 h, cells in the upper part were removed and migrating cells were loaded with DAPI fluorescent stain. Migrating cells were then imaged by fluorescence microscopy and quantified using a semi-automated system, with the ImageJ software.

### 4.8. Drug Combination Studies

Studies of NO-NSAIDs combined with erlotinib were conducted to evaluate the difference between their combined effects and theoretical additive effects, according to the Loewe model. Viability was evaluated at 96 h by MTT reduction and data were analyzed using the COMBENEFIT software [32].

### 4.9. Statistical Analyses

Except for those from drug combination studies, data were analyzed using GraphPad Prism Software (V 7.0, GraphPad Software, San Diego, CA, USA). EC_50_ values were obtained by fitting the data to the four-parameter equation. All data are expressed as the mean ± SD of at least three independent experiments. One-way ANOVA was performed to compare groups, using Dunnett’s post-test to compare data with the control group, or Tukey’s post-test to perform comparisons between all experimental groups. For all comparisons, a *p*-value of 0.05 was considered the significance threshold. 

## 5. Conclusions

In this study, we demonstrated that NCX4040 was more potent than NCX4016 and aspirin in decreasing NSCLC cell viability and migration of NSCLC cells. Also, NCX4040 exhibited significant synergistic effects in combination with erlotinib (an epidermal growth factor receptor inhibitor) in erlotinib-resistant cells. C-PTIO (an NO^•^ scavenger) reversed the antiproliferative effects of NCX4016, but not those of NCX4040. Finally, misoprostol (a PGE_2_ receptor agonist) significantly reversed the antiproliferative and antimigratory effect of NCX4040, but not those of NCX4016. This report suggests that the PGE_2_ inhibition is important in the mode of action of NCX4040, shedding new light about the pharmacological differences between the different NO-aspirin isomers.

## Figures and Tables

**Table 1:** Effect of NO-aspirins on the viability and proliferation of non-small-cell lung cancer (NSCLC) cells.

	EC_50_ (µM) ^1^	
	MTT	BrdU
Aspirin	937.7 ± 162.3	2,534 ± 142.1
NCX4016	83.0 ± 5.4 ****	101.0 ± 15.6 ****
NCX4040	13.5 ± 2.6 *****	7.8 ± 1.3 ****

^1^ The EC_50_ values shown are the mean ± standard deviation of three experiments, each performed in triplicate. **** *p* < 0.0001, compared with aspirin, calculated by one-way ANOVA and Dunnett’s post-test.

**Table 2:** Equipotent concentrations for erlotinib, aspirin, NCX4040, and NCX4016 in three NSCLC cell lines.

	NSCLC Cell Line (EC_50_) ^1^		
	A549	H1299	H1975
Erlotinib	8.22 µM	12.1 µM	4.77 µM
Aspirin	0.993 mM	1.14 mM	1.11 mM
NCX4040	237 µM	93.1 µM	202 µM
NCX4016	23.8 µM	13.8 µM	7.0 µM

^1^ EC_50_ is the concentration that reduces cell viability by 50%, normalized using control data. Cells were exposed to different drugs in two-fold dilution series for 96 h. Data were loaded in COMBENEFIT to plot concentration–response curves.

**Table 3:** Maximal synergy scores for the combination of erlotinib with aspirin, NCX4040, or NCX4016 in three NSCLC cell lines.

	Max Synergy Score by Cell Line ^1^		
	A549	H1299	H1975
Erlotinib + aspirin	7 ± 5 *	3 ± 0 *	5 ± 1 *
Erlotinib + NCX4040	20 ± 3 *	13 ± 3 *	4 ± 1 *
Erlotinib + NCX4016	9 ± 2 *	6 ± 6	6 ± 1 *

^1^ Synergy scores represent the difference between the real data obtained and the synergy model obtained after loading data in COMBENEFIT, which builds an XYZ model of the combination, using the effect of each drug alone and the Loewe’s model of drug additivity. Differences between the theoretical combinations and empirical data are represented by a number generated for each combination point. Positive increasing values indicate the extent of synergy. Data are presented as the mean ± standard deviation of three independent experiments, each performed in duplicate. * *p* < 0.05, compared with the Loewe’s additivity model, calculated by *t*-test.

## Supplementary Materials

The following are available online. Figure S1: Effect of NO-aspirins on the viability and proliferation of non-small-cell lung cancer (NSCLC) cells; Figure S2: Concentration–response curves of aspirin, NO-aspirins, and erlotinib on viability of non-small-cell lung cancer (NSCLC) cells; and Figure S3: Effect of NO-aspirins combined with erlotinib on the viability of non-small-cell lung cancer (NSCLC) cells.
