# Supplementary material for: Reconsidering the Role of Cyclooxygenase Inhibition in the Chemotherapeutic Value of NO-Releasing Aspirins for Lung Cancer
Source: Molecules. 2019 May 18;24(10):1924. doi: 10.3390/molecules24101924 (PMC6572483; doi:10.3390/molecules24101924)
Supplement: Supplementary file 1 [file molecules-24-01924-s001.pdf]

molecules

Supplementary material

Reconsidering the role of cyclooxygenase inhibition in the chemotherapeutic value of NO-releasing aspirins for lung cancer

Antonia Martin-Martin[1], Andrés Rivera-Dictter[1], Matías Muñoz-Uribe[1], Freddy Lopez-Contreras[1,2], Jorge Perez-Laines[1], Alfredo Molina-Berrios[3] and Rodrigo López-Muñoz[1*]

1 Instituto de Farmacología y Morfofisiología, Facultad de Ciencias Veterinarias, Universidad Austral de Chile. Valdivia, Chile; (rodrigo.lopez@uach.cl, R.L-M)
2 Escuela de Graduados, Facultad de Ciencias Veterinarias, Universidad Austral de Chile. Valdivia, Chile; (freddy.lopez@postgrado.uach.cl, F. L-C)
3 Instituto de Investigación en Ciencias Odontológicas, Facultad de Odontología, Universidad de Chile. Santiago, Chile; (aemolina@u.uchile.cl, A.M-B)
* Correspondence: rodrigo.lopez@uach.cl Tel.: +56-632-444321. (R. L-M)

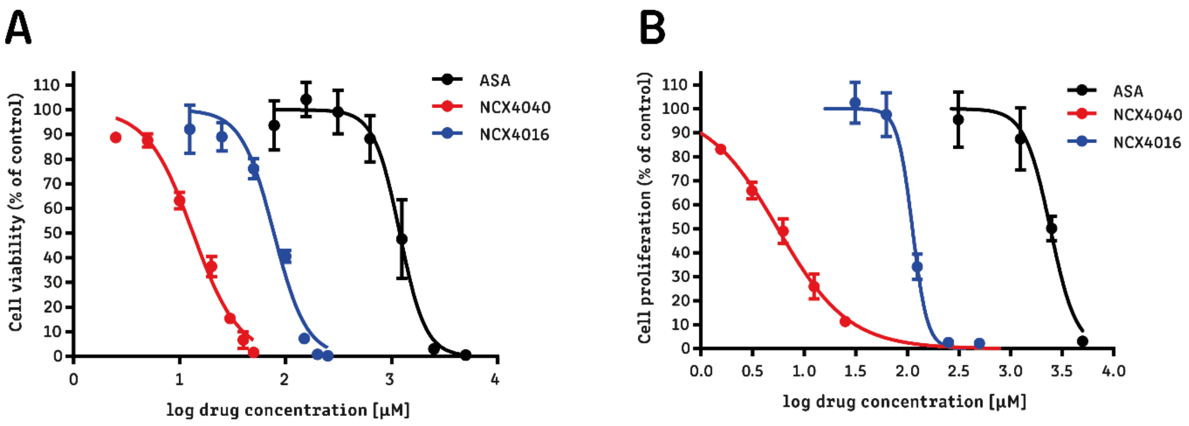

Supplementary Figure S1. Effect of NO-Aspirins on the viability and proliferation of non-small cell lung cancer (NSCLC) cells. H1299 cells were exposed to aspirin, NCX4016, and NCX4040 in 2-fold dilution series. Cell viability (by MTT reduction) and proliferation (by BrdU uptake) were measured at 96 and 24 h, respectively. **A.** Effects of aspirin (ASA), NCX4016, and NCX4040 on H1299 cell viability. **B.** Effects of aspirin (ASA), NCX4016, and NCX4040 on proliferation of H1299 cells. Graphs show representative graphs of three experiments performed in triplicate.

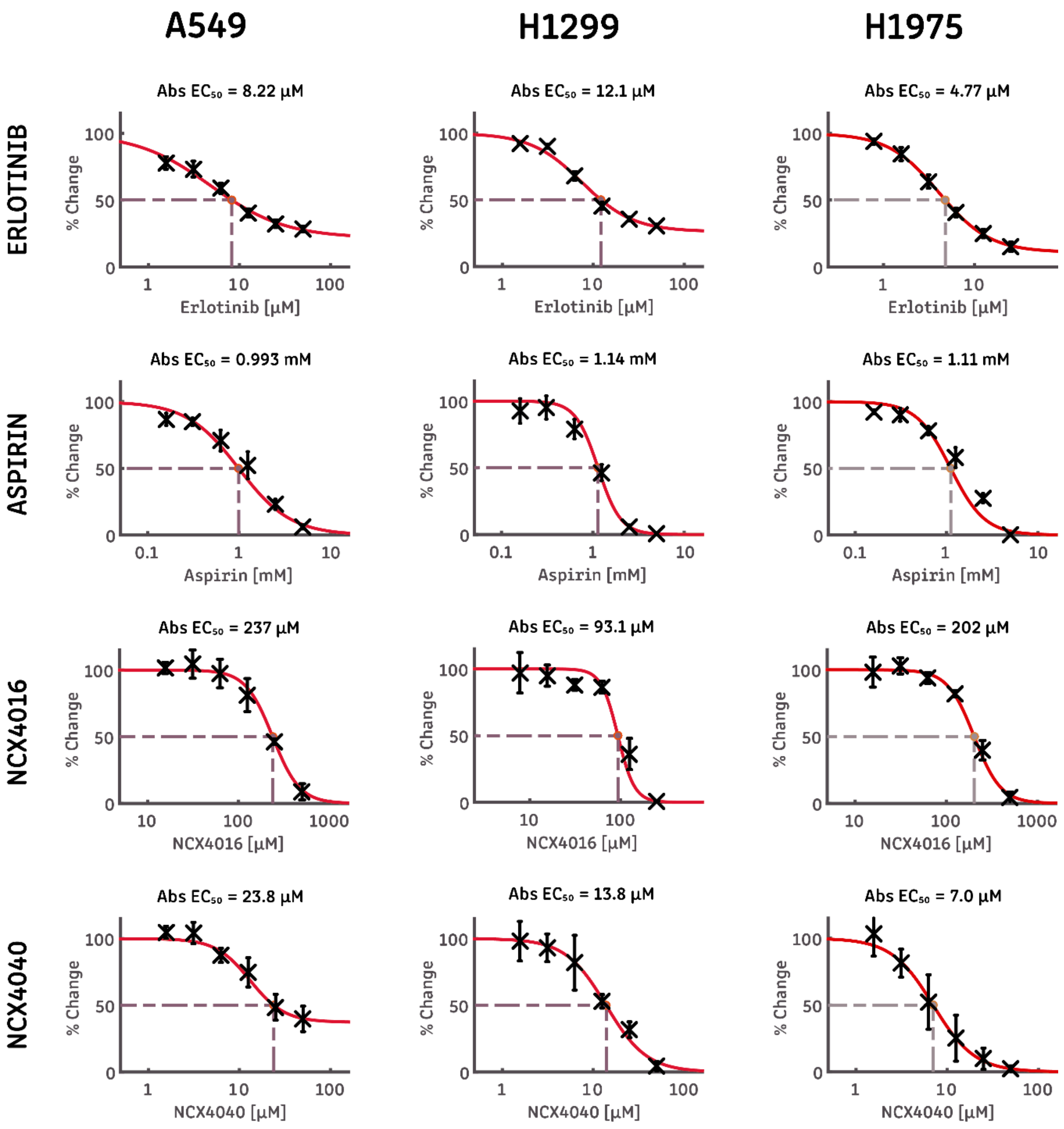

Supplementary Figure S2. Concentration-response curves of aspirin, NO-Aspirins, and erlotinib on viability of non-small cell lung cancer (NSCLC) cells. The effects of aspirin, NCX4016, NCX4040, and erlotinib on cell viability were measured by MTT assay. Three NSCLC cell lines were used: A549 (left column), H1299 (middle column), and H1975 (right column). Cells were exposed to different drugs in 2-fold dilution series for 96 h. Data were loaded in COMBENEFIT software to plot concentration-response curves and combination studies (Figure 4). “Abs EC_{50}” indicates the concentration that reduced cell viability by 50%, normalized using control data.

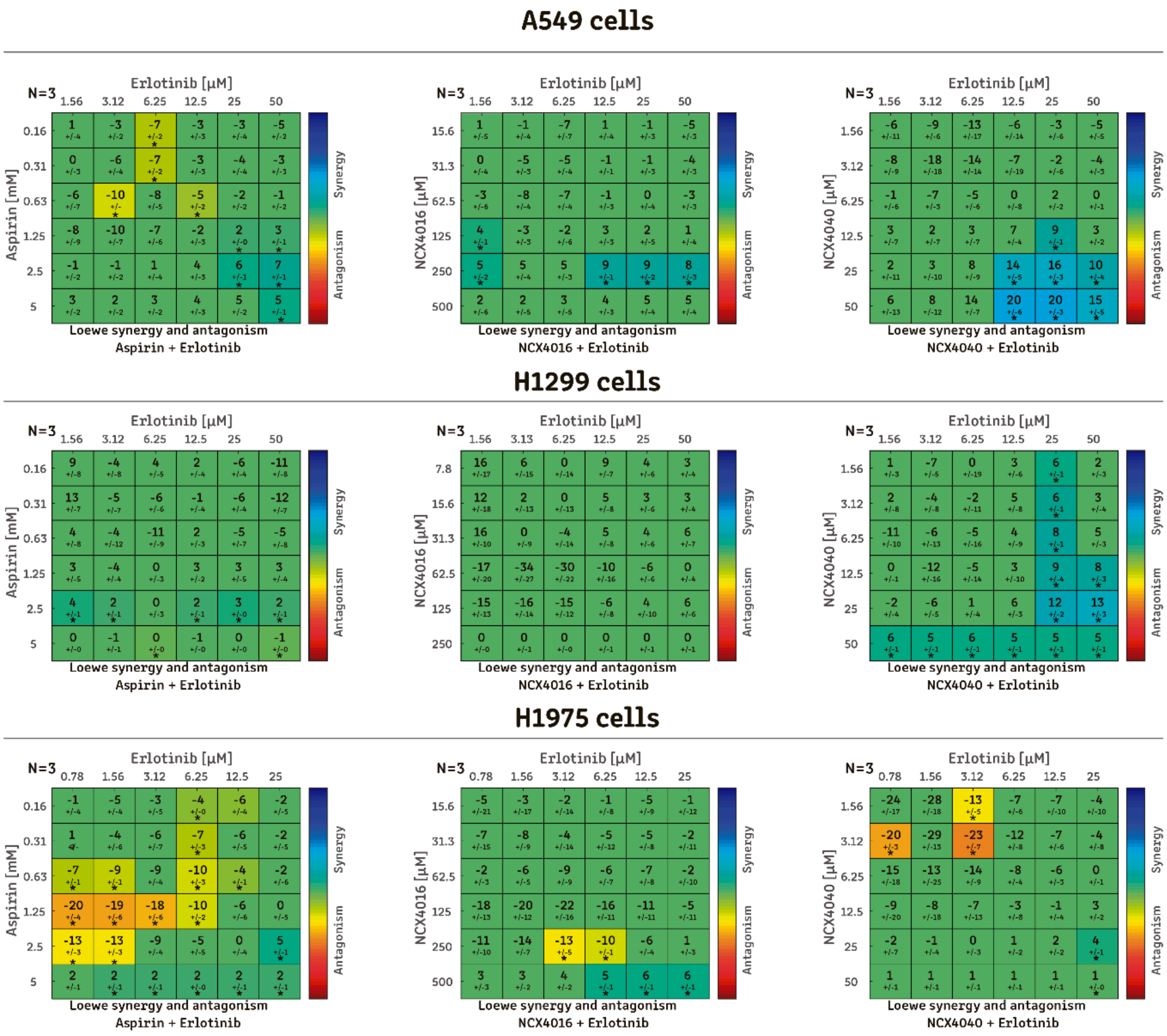

Supplementary Figure S3. Effect of NO-Aspirins combined with erlotinib on the viability of non-small cell lung cancer (NSCLC) cells. The effects of combinations of aspirin, NCX4016, and NCX4040 with erlotinib on cell viability were measured by MTT assay, using three NSCLC cell lines: A549 (upper panels), H1299 (middle panels), and H1975 (lower panels). Cells were exposed to the different drugs for 96 h. For drug combinations, two-fold dilution series, comprising six concentrations, were mixed in every possible combination. Effects matrices were plotted using COMBENEFIT software, which builds an XYZ model of the combination, using the effect of each drug alone, and the Loewe's model of drug additivity. Differences between the theoretical combinations and empirical data are represented by a number generated for every combination point. Positive numbers represent synergistic combinations, while negative numbers indicate antagonistic interactions. The color code shows combination points that are significantly different from the theoretical model, calculated by t-test. *$p < 0.05$. Each graph represents the mean ± standard deviation of three independent experiments, each performed in duplicate.

© 2019 by the authors. Licensee MDPI, Basel, Switzerland. This article is an open access article distributed under the terms and conditions of the Creative Commons Attribution (CC BY) license (http://creativecommons.org/licenses/by/4.0/).
